# Mechanisms through which befriending services may impact the health of older adults: A dyadic qualitative investigation

**DOI:** 10.1177/13591053241235846

**Published:** 2024-03-04

**Authors:** Caoimhe Hannigan, Michelle Kelly, Eimile Holton, Brian Lawlor, Thomas Scharf, Frank Kee, Sean Moynihan, Aileen O’Reilly, Joanna McHugh Power

**Affiliations:** 1 National College of Ireland, Ireland; 2 Maynooth University, Ireland; 3 Trinity College Dublin, Ireland; 4 University of Newcastle, UK; 5 Queen’s University Belfast, UK; 6 ALONE, Ireland

**Keywords:** healthy ageing, intervention, loneliness, social support, subjective wellbeing, volunteering

## Abstract

Befriending services are often delivered to older adults with a view to improving social connectedness, but they may also lead to improved health. The objective of the current study was to explore potential mechanisms through which befriending services might impact the health of older adults. Data were collected from 13 befriendee-befriender dyads (*n* = 26), using a constructivist grounded theory and dyadic analytic approach. Potential mechanisms were described, using a realist evaluative framework of mechanistic processes in a complex intervention context. Five mechanisms of action triggered by the intervention were identified: supporting health behaviours; providing emotional support; improving mood; getting cognitive stimulation and novelty; and providing opportunities for socialising. We identified five potential mechanisms through which befriending services might impact health for older people. Our results suggest potential mechanisms through which befriending might positively impact the health of older people, and which should be evaluated empirically in future research.

## Introduction

Befriending interventions provide supportive relationships on a voluntary basis via regular visits or phone calls, most typically to those experiencing social isolation and loneliness (Dean and Goodlad, 1998; Gardiner et al., 2018; Siette et al., 2017). Little evidence exists that befriending interventions actually alleviate loneliness (Beckers et al., 2022; Cattan et al., 2005; Krohne et al., 2022). In the context of such lack of evidence, Age UK have expressed concerns about the application of befriending interventions to the problem of loneliness (Jopling, 2015).

However, befriending interventions might yield benefits other than reducing loneliness. A group-based, long-running Finnish intervention, Circle of Friends, has demonstrated positive effects on cognitive function (Pitkala et al., 2011), psychological wellbeing (Routasalo et al., 2009), mortality, subjective health and healthcare utilisation (Pitkala et al., 2009). A structured peer-to-peer support program, meanwhile, failed to demonstrate any effect on physical health (Schwei et al., 2021), although methodological limitations may have occluded the detection of an effect. Similar issues with methodological quality or design have been highlighted elsewhere in the befriending literature (Siette, et al., 2017). With respect to mental health, befriending interventions yield a therapeutic effect on depressive symptoms and emotional distress (Mead et al., 2010) (but see criticisms of Mead’s conclusions; El-Baalbaki et al., 2010), psychiatric symptoms (Sikira et al., 2021), and can enhance emotional wellbeing (Moss et al., 2021)

There is a rationale for expecting a positive effect of befriending on health, defined as ‘a state of complete physical, mental, and social wellbeing, and not merely the absence of disease or infirmity’ (World Health Organization, 1995). Elective relationships have a positive impact on health, and it could therefore be expected that these benefits might also be seen in the context of befriending relationships (Berkman et al., 2000; Golden et al., 2009). Berkman’s causal cascade model, while more broadly about the impact of social contexts on health, may also offer a theoretical framework through which to understand the various ways in which social connection (potentially encompassing that offered by befrienders) could impact health in later life (Berkman et al., 2000). For instance, in Berkman’s model, one could draw a pathway from informational social support (which could theoretically be delivered in a befriending context) to health behavioural pathways to improved health (e.g. quitting smoking, getting exercise).

As such there is limited but growing evidence that befriending is good for health including mental health. We may further clarify how befriending interventions might impact mental and physical health by considering mechanisms of action, which is a priority for such complex interventions as befriending (Fakoya et al., 2021) (Bonell et al., 2022; Moore et al., 2014). Inspection of such mechanisms is typically a task left relatively neglected by qualitative researchers exploring befriending and its impact (Balaam, 2015; Fakoya et al., 2021), and such neglect has been cited as the root cause of a broader lack of understanding of how befriending impacts older adults (Gardiner and Barnes, 2016). Qualitative approaches are capable of yielding critical insights into potential mechanisms underlying complex interventions, by allowing direct engagement with intervention recipients (Bonell et al., 2022). In other words, qualitative research goes beyond asking ‘what works’ to asking ‘how it works’ (Godfrey, 2015). Intervention mechanisms can be defined as ‘new human responses, actions, and interactions triggered by the provision of new economic, informational or other resources’ (Bonell et al., 2022: 2). Per Bonell’s description, mechanisms are not directly observable but affect some outcome (in our case, the outcome of interest is improved health).

Previous research has explored the mechanisms through which befriending might impact loneliness and mental health. Two studies have looked at potential mechanisms through which befriending impacts loneliness and social isolation (Fakoya et al., 2021; Krohne et al., 2023). Another qualitative study explored potential mechanisms through which befriending services might improve wellbeing at end of life, and demonstrated that befrienders might impact wellbeing by providing meaningful interactions, a sense of connectedness, supporting family members, and cognitive engagement (Gardiner and Barnes, 2016). However, empirical evidence for such mechanisms in the link between befriending interventions and health is lacking.

As such, the aim of the current study was to build a theory of the mechanisms through which befriending might impact health, by interviewing the older person users and the voluntary providers (‘befrienders’) of a befriending service. While previous studies of befriending have similarly interviewed both members of the befriending ‘dyad’ (Bantry-White et al., 2018), the older person user and the befriender, they have not explicitly compared dyadic input (Breheny et al., 2020). Comparing the views of befrienders and the older persons who use the service is likely to provide a more comprehensive understanding of how befriending may impact the health of older users.

We used the 32-item COREQ checklist and the American Psychological Association journal article reporting standards for qualitative research (‘JARS-qual’) to structure our reporting (Levitt et al., 2018; Tong et al., 2007).

## Methods

### Theoretical framework and study design

The study was conducted as the qualitative arm in a parallel convergent mixed-methods study (citation masked for review). A constructivist grounded theory framework was used (Charmaz, 2006) coupled with principles from dyadic analysis (Eisikovits and Koren, 2010). Grounded theory is a methodology which uses a highly systematic inductive approach to building a theory which is ‘grounded’ in data (Glaser and Strauss, 2009); constructivist grounded theory, meanwhile, is more relativist than traditional grounded theory, emphasising the active role of the participant in constructing their own reality and encouraging the integration of reflexive and contextual information in the development of a grounded theory (Charmaz, 2006). Constructivist grounded theory is particularly suitable when trying to understand the mechanisms of action in interventions related to health (Hunter et al., 2016; Yu and Liu, 2010). Grounded theory with dyadic analysis has previously been used to effect with populations such as later-life re-partnered couples (Koren, 2016).

Dyadic analysis, meanwhile, is the analysis of data from interviews with any dyad or pair of interest, and is compatible with other traditions of qualitative analysis, including grounded theory analysis. Dyadic analysis can create a deeper understanding of a relationship since it permits triangulation by comparing the perspectives of both members of a dyad on the relationship (Eisikovits and Koren, 2010). According to Eisikovits and Koren (2010), individual interview data are desirable to supplement dyadic joint interviews, since participants may feel constrained by the presence of the other dyad member if only dyadic interviews are conducted. The dyadic approach is suitable in research concerning couples or pairs, such as the relationship between the befriender and the older person who is using the service (hereafter referred to as the older person). As such, since we wanted to explore a topic which concerned an existing dyad, dyadic analysis is appropriate.

### Context, recruitment and sample

In Ireland, befriending services are provided by a range of voluntary and non-governmental organisations, and have been networked by ALONE under the Community Impact Network (https://alone.ie/community-impact-network/). ALONE is a nongovernmental organisation dedicated to supporting the lives of vulnerable older adults. Their befriending service involves non-structured, weekly visits from trained matched volunteers to the homes of older people. Befrienders are matched for age, gender and location to older persons, and older persons and befrienders can request a rematch if they feel there is little rapport. Beyond providing training to volunteers, which encourages weekly visits of 1 hour duration, there is no surveillance of the types of engagement or number of interactions (see Burke, 2015).

Through COVID-19, befrienders we spoke to in this study continued their relationships with their matched older person via the phone or visits outdoors. As part of a broader evaluation of the impact of the ALONE befriending service on new older person users, we also recruited existing older persons using the ALONE befriending service, using a critical case sampling design, which is highly purposive and thus high in rigour. User-befriender dyads were the unit of recruitment. Estimating sample size in a grounded theory context is always challenging (Charmaz, 2006). We employed the informational power approach to sample size estimation (Malterud et al., 2016), which requires consideration of: specificity of study aim (here: specific); sample specificity (here: very specific, since critical case sampling was used); use of theory (here: no); dialogue quality (here: expected to be moderate due to lack of prior relationship with interviewers); and analytic strategy (here: cross-case). Judgements made using this framework yielded low to moderate sample size requirements, taking into account the research team’s prior experiences in conducting research with similar populations of older adults, so a sample of 10–15 dyads was agreed. Inclusion criteria for recruitment were that dyads had to be in existence for at least 1 year as a pair; living within the greater Dublin area (this was later relaxed); and capable of providing informed consent to participate in the research. Initially, all interviews were planned in person, but with the advent of COVID-19, interviews following Dyad 3 were all done by phone, meaning that participants were then recruited from all over Ireland (since the geographical inclusion criterion was a practical one, designed to limit the amount of travel necessary by the interviewer). Ultimately, interviews were completed with 13 dyads. A staff member in ALONE recruited all participants and, with consent, passed their details to the research team, who then underwent the process of informed consent with all participants. The study was approved by the local Research Ethics Committee at the Faculty of Health Sciences, Trinity College Dublin. In total, 15 dyads had their details passed to the research team—all 15 contacted by the staff member at ALONE consented to having their details passed on. Due to non-response, interviews were ultimately conducted with 13 dyads.

### Data collection and setting

Two interview guides, one for the older persons and one for the befrienders, were developed according to constructivist grounded theory guidelines (Charmaz, 2006). That is, we began with sensitising concepts from our literature review as tools for encouraging our participants to reflect on a purposively narrow question: how befriending might impact health. We designed the schedule to elicit an in-depth reflection using open-ended and non-judgemental questions. Whether interview schedules in constructivist grounded theory are highly structured or more loose depends on the skill level of the researcher; we kept ours moderately structured but flexible depending on incoming data. Following Charmaz’ recommendations (2006), we used an initial list of open-ended, contextualising questions, followed by intermediate questions which probed the specific question of how befriending might impact health, followed by some wrap-up questions eliciting any other feedback the participant may have which was not covered by the preceding schedule. The intermediate questions were based on sensitising concepts including: first impressions of the older person; changes in the older person’s physical and mental health; changes to the befriending relationship and visits; perspectives on the impact of befriending on health; changes desired to the service; lessons learned from being a befriender. The interview guide for older persons focused on the following areas: life before service uptake; their experience of loneliness; managing wellbeing; how they started to get the service; expectations of the service; impact of the befriender on them and their lives. Prior to the interview each participant was sent an information sheet and consent form which they returned if interested in engaging in the study. The first dyad interviewed yielded very short interviews (4 and 11 minutes respectively) which was subsequently judged to be an issue in researcher training, so the data from these interviews was not retained for analyses. Aside from the first two included dyads, each interview was conducted on the phone and lasted between 18 and 59 minutes (see Table 1).

**Table 1.** Sample characteristics of *n* = 12 dyads.

Dyad number	Role	Age	Gender	Date of interview	Duration of interview (minutes)	Duration of befriending relationship
2	Older person	82	Female	23/5/19	19	2 years
2	Befriender	43	Female	11/6/19	32	2 years
3	Older person	69	Female	22/1/20	53	3 years
3	Befriender	36	Female	29/1/20	36	3 years
4	Older person	74	Female	18/4/20	45	15 months
4	Befriender	58	Female	16/4/20	40	15 months
5	Older person	86	Female	05/06/20	39	2 years
5	Befriender	47	Female	05/06/20	25	2 years
6	Older person	86	Male	22/06/20	61	18 months
6	Befriender	35	Male	12/06/20	48	18 months
7	Older person	81	Female	07/07/20	46	2 years
7	Befriender	24	Female	21/07/20	18	2 years
8	Older person	76	Male	13/10/20	39	1 year
8	Befriender	59	Male	4/09/20	44	1 year
9	Older person	78	Male	03/11/20	74	2 years
9	Befriender	42	Male	02/11/20	25	2 years
10	Older person	71	Female	25/01/21	41	3 years
10	Befriender	37	Female	17/11/20	32	3 years
11	Older person	69	Female	19/01/21	37	1 year
11	Befriender	24	Female	26/11/20	20	1 year
12	Older person	79	Female	17/12/20	59	2 years
12	Befriender	39	Female	27/11/20	24	2 years
13	Older person	96	Female	15/12/20	20	1 year
13	Befriender	45	Female	01/12/20	26	1 year

For the first three dyads, individual interviews and a joint interview (i.e. with both members of the dyad present in a single interview) were conducted, all in person. However, the arrival of the COVID-19 pandemic and associated public health guidelines implemented in Ireland in March 2020 meant that face-to-face interviews were no longer possible. The decision was made to limit the data collection for the remaining ten dyads to separate phone-based interviews for each dyad member (from dyad 4 onwards), as most older participants were unable to engage in online interviews and conference calls would yield poor data in this context. Although we conducted dyadic interviews with dyads 2 and 3, only their individual interview data were pooled for analysis (to ensure consistency with the remaining dyads). The dyadic analysis of individual interviews is in fact exactly what was advocated by the original authors of the approach (Eisikovits and Koren, 2010) on the grounds that they enable each participant to tell their own story, and that supplementing such individual interviews with a joint interview might negatively ‘affect the benefits of both the separate and the joint interviews’ by changing the audience (Eisikovits and Koren, 2010: 1643).

### Rigour and reflexivity

We undertook strategies to ensure rigorous, reflexive research was conducted. We developed a rigorous conceptual framework in which to position the research aim. Data collection was undertaken by multiple individuals and transcripts were analysed by multiple individuals—two for each transcript—(authors JMcHP, CH, EH)), to ensure triangulation and to enable the calculation and resolution of inter-rater agreement. In keeping with the principles of constructivist grounded theory, we analysed the transcripts from each dyad immediately after data collection and prior to collecting data from the next dyad, so inter-rater agreement was calculated at each interim point, and was never below 80%. When differences were found in the analyses conducted by the two raters of each transcript, the results were discussed until a resolution was reached. Triangulation was further permitted through the dyadic analytic technique, which allowed the research team to compare and contrast findings across members of a dyad.

Authors JMcHP and CH (both academic psychologists) were trained to doctoral level and had substantial prior experience of research interviews, while author EH (a research assistant) was trained to Masters level and had received training to conduct the interviews. Interviewers did not interact with the dyads prior to the interviews other than to arrange the interview by phone. All three interviewers were psychologists working in the field of gerontological research and academic psychology, and all were involved in an evaluation of the befriending service (led by author JMcHP as Principal Investigator, and CH as co-investigator). Continued discussion making use of memos between the primary researchers (JMcHP, CH, EH) facilitated development of the themes.

Trustworthiness of the study findings was of concern throughout the study (Lincoln, 2005). Transcripts were typed verbatim from audio recordings of all interviews, thus improving the study credibility; sample characteristics and procedure were reported to allow readers to gauge transferability and dependability of results; and the reflexive practices of the research team (discussion, memos) worked to promote the confirmability of the study results.

While member checks were not employed in this study, due mostly to the difficulties of phone-based data collection during COVID-19, we presented the study results to a group of befriending service users at the end of the study in a half-day workshop, and discussed the results with them. The aim of this workshop was to sense-check study results, clarify potential misunderstandings, and discuss potential applications of the findings. The workshop was recorded using field notes taken by a research assistant, and the research team then reviewed their interpretation of the study results, the final version of which are presented below.

Data availability statement: we did not seek consent from participants to archive their data, so regrettably there is no way to share the data from this study. We wrote the ethics application for this study in 2018 and since have always sought to archive data.

### Analysis

We used a constructivist grounded theory analytic method (Charmaz, 2006) informed by principles of dyadic analysis (Eisikovits and Koren, 2010). While interviews were conducted separately, analysis was conducted dyadically. As per grounded theory principles, the first step of line-by-line initial coding was completed with each interview transcript, followed by a process of focused coding, with a final step of theoretical coding. Initial codes were retained until they were no longer useful in relation to the emerging theory. Codes were retained only if they arose from multiple dyads. Then, comparisons between members of each dyad were made and overlaps and contrasts noted. To ensure the analysis was trustworthy, audio-recorded interviews were first transcribed verbatim, analysed by two researchers independently, and a process of peer debriefing was used with the co-authors of this manuscript and with a Patient and Public Involvement (PPI) committee established at the beginning of the study. As per guidelines (Koren, 2016), data were presented per theme with quotations from each partner of a dyad, further enhancing study rigour.

## Results

**Figure 1.**
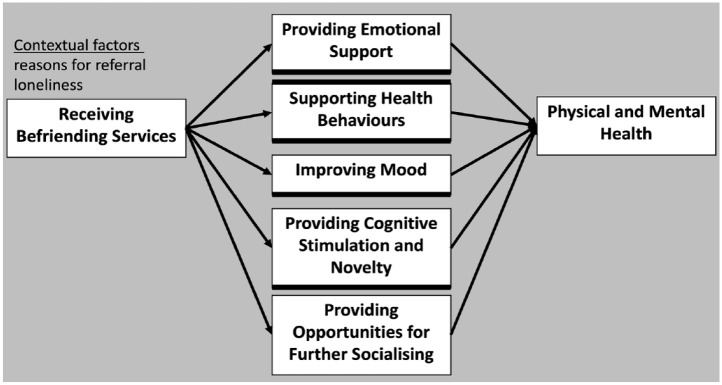
A grounded theory of five potential mechanisms through which befriending may impact the health of older adults, with respect to contextual factors identified in the study.

The two samples were described in Table 1. Five intervention mechanisms were identified: supporting health behaviours (exercise, nutrition, access to healthcare); providing emotional support; improving mood; getting cognitive stimulation and novelty; providing opportunities for socialising (see Figure 1). Participants discussed how these mechanisms operated in the context of pre-intervention health and loneliness.

### Contextual factors

Some older persons had sought or been referred to the befriending service, for reasons of loneliness (OP2, 3, 4, 9) depression (OP15), and physical illness (OP5, 6, 10, 11). Physical illness was, for some, causing or at risk of causing isolation, leading to referrals, OP10: ‘I was actually in hospital . . . they told me they were putting me in contact with ALONE’.

Older persons also mentioned their pre-intervention mobility issues (OP2, 3, 5, 9, 10), chronic health issues (OP4), and mental health issues (OP10, OP15). Dissonance within the dyad was clear for some, for example, Dyad 4, where OP4 mentioned that ‘my health is not so bad . . . I’d say I’m healthier than maybe a lot of people my age’ while BF4 commented ‘She’s not in the best of health . . . I don’t exactly know what her health problems are’. However this dissonance may be due to broader negative perceptions of ageing and its impact on health, as described among befrienders previously (Breheny et al., 2020).

Another critical contextual factor was the pre-intervention loneliness experienced most older people interviewed, likely exacerbated by the COVID-19 pandemic and associated social distancing measures in place in Ireland during 2020 and 2021, for example, OP13: ‘I was lonely, yeah. Well, living on your own is very lonely’. BF13 also felt that OP13 was very lonely because of the lack of visitors during the pandemic: ‘She is very lonely even now, well, currently with the way the situation is, very lonely, very, very lonely. ‘Cos there’s not as many people calling down’. Almost all participants reported feeling lonely, and one befriender claimed that the intervention service would relieve such loneliness: for example, BF8: ‘Sometimes older people settle into their loneliness but we’re probably taking them out of it a little bit more’.

In such contexts, then, five mechanisms were identified as being triggered by the befriending intervention and likely to yield positive impacts on health.

#### Mechanism 1: Supporting health behaviours

Based on our data, we posit that befriending services may improve health through the mechanism of support from befrienders in relation to the older person’s engagement in health behaviours, such as exercise engagement and healthy nutrition. Some befrienders had a clear active role in supporting the older person’s health, sometimes by informational support and encouragement. For some of the older persons, the befriending visit was an opportunity to take exercise safely, since many had issues with stability and mobility, for example, BF13 described some exercises she did with OP13: ‘I was showing her a few exercises in the chairs, they were chair exercises, and she said but I’d need you here all the time; I couldn’t do them on my own’.

Befrienders also had a role to play in nutritional support. Two befrienders described helping their partners to get groceries: OP15: ‘she brings me this time more food, you know? She say what you want ‘cos I will bring, sometimes I say nothing, but sometimes she buys more!’/BF15: ‘I think I helped her out buying the stuff, I think she found all that really good as well . . . what I’ve done really is brought her to the supermarket or brought her to the shops if she needed something’.

Befrienders also facilitated access to healthcare. BF7 made herself available to bring OP7 to the hospital for appointments: ‘I suppose she may, like if she couldn’t get a lift up to the hospital she may not have gone to the hospital for that visit if you get what I mean’. BF7 noted that OP7 became more comfortable with her over time, and more able to ask for favours like lifts to hospital appointments: ‘She would have kind of come out of her shell a little bit more and as we got to know each other . . . she’d be a lot more open . . . to ask maybe if she needed to go to the hospital or an appointment . . . I have no problem taking her’.

Sometimes healthcare access involved visiting the chemist particularly during the COVID-19 pandemic, for example, OP10: ‘BF10, as I said, was a little angel. So, she went to the chemist for me when I needed and things like that. But I hated asking her to do anything for me’/BF10: ‘I mean she knows I’m right down the road from her and I’m always saying please let us know if you need anything’.

#### Mechanism 2: Providing emotional support

Based on our data, we suggest that befriending services may play a role in supporting health through the mechanism of emotional support provided by befrienders for the older persons. Many dyads stated that befrienders became confidantes for their partners, providing emotional support for them, particularly in the context of family difficulties. Dyads differed in the extent to which they shared personal information with each other. For some, the chat was kept light, for example, OP7: ‘we just sit and chat and we watch “Say Yes to the Dress” and criticise everybody’/BF7: ‘you’d visit and you’d have a cup of tea, and then after a while it got to where we knew which biscuits each other liked and we’d have brought them’. In one dyad, the befriender would typically confide in the older person, rather than the other way around: OP5: ‘I wouldn’t be talking very much about personal things’/BF5: ‘We would chat about different things to do with my personal life and it’s, not that it’s a bit of therapy but it is at the same time you know? . . . she has no problem giving you that little bit of advice’.

Many dyads reported confiding in each other about family issues, such as OP12: ‘BF12 is a very good listener and she tells me, you know, she put a different slant on things I mightn’t think about, you know? Especially as things are, she knows family trouble here now . . . she’s like a counsellor! . . . She’s gone through the tears and everything with me’/BF12: ‘I think at times it’s like she has a safe space or she’s able to tell me even that she’s going to counselling, whereas some of her family may not know things are happening . . . I suppose she didn’t feel like she even had a space to just talk about what was happening for her . . . she really was very isolated in that regard’. BF12 later added that she was grateful that OP12 had sought counselling; ‘I think it was really important that she got her separate counselling as well because I’m conscious of my role in it too . . . you don’t want to disempower somebody’.

#### Mechanism 3: Improving mood

Based on our data, we propose that befriending services may improve health partly by improving the mood of older persons. Central to many interviews was the fun involved in the befriending visits, and some older people felt that this improved their health, for example, OP8: ‘BF8 being jolly and happy is, and having a joke is a good thing too. You know, they say laughs are the best medicine’; OP8: ‘we do play cards, we have a very competitive game . . . we’d have a good laugh anyway’.

OP7 felt that befriending improved her mood, rather than her physical wellbeing: ‘I mean it hasn’t taken the rheumatoid away or anything like that but it does mentally, mentally, I mean if I′m having a bad day and I know that BF7 is coming, for example she’s coming this evening and I look forward to her coming, she’s a very positive thing in my life’. BF7 also described that she felt able to improve the mood of OP7 up if needed: ‘she might be a bit down and you can sense that she might be down when you go in on a Tuesday evening or whatever but you kind of know how to work it . . . if she’s in bad form you kind of know how to perk her up a wee bit’. OP3 described the impact that her befriender had by saying ‘When BF3 came she brought me out of myself’.

#### Mechanism 4: Providing cognitive stimulation and novelty

Based on our data, we propose that befriending services may improve health of older persons, specifically their cognitive health, by providing cognitive stimulation.

Many dyads described the ways in which the befriending partnership introduced stimulation and novelty to the lives of the older person. OP3 for instance referred frequently to the amount that she learned from her befriending partner: ‘BF3 has educated me in a way . . . I think if she were an older person, we’d just be talking about our kids and grandkids’. Some dyads highlighted the content of their conversations, which often concerned shared interests or current affairs. For OP4, the befriending visits were mostly about chatting, whereas BF4 described these chats as being educational: ‘I like just having someone to talk to every week and someone to invite into my home’/BF4: ‘we’ve great chats, she’s very well read, very highly educated, so there’s nothing we don’t discuss’. BF8 referred, in response to a question about whether his relationship impacted OP8’s health, by referring to cognitive stimulation: ‘I think I can keep him engaged’.

Similarly, for some pairs, the opportunity of an intergenerational exchange provided its own novelty to older persons. For instance, OP3: ‘I think what amazes me she’s so young. And I’m 69 nearly. To me that, to have that friendship with someone that age, it’s fantastic’/BF3: ‘It’s a real enhancement because I think that we all tend to fall into step with the people we’re in college with, the people we work with -our own demographic’. Both OP3 and BF3 reported that they had a true friend in each other, meaning that the befriending service had yielded a true friendship. Similarly, OP12 was very interested in hearing about her partner’s life: OP12: ‘I look forward to the visit now. It’s the younger person’s view’.

#### Mechanism 5: Opportunities for further socializing

Based on our data, we propose that the befriending service itself may improve health through offering further opportunities for social engagement. Interestingly, the organisation itself may have also provided some social context. One service the participants spoke highly about was the ‘check-in’ phone call some of them had received, OP3: ‘there’s another girl from the office rings you every now and then, to ask you are you happy with your relationship with your companion that comes. So at any time you can change your companion, you know, if you don’t see eye to eye with someone’. Her befriender had an additional insight into the value of the service for OP3, BF3: ‘She likes the fact that there’s now somebody else in her life, and when I say somebody else, I mean another organisation, another institution, another thing to identify with and ally herself to. There’s another element of connectedness’. Participants spoke frequently about the events organised by ALONE as being highlights, for example, BF15: ‘the events were something I know I would have enjoyed pre-COVID, so I think things like that people really like and appreciate and get a lot out of so I think they’ve been brilliant at doing that stuff as well before COVID, but I think eventually they’ll be able to go back to doing those things’; OP4: ‘I go and I meet the ALONE people, I go and have lunch, an ALONE lunch, once a month in a hotel, it’s meant to be really part of the cinema club outing, but I don’t go to the cinema I just go to lunch and a few other people do the same thing. And it’s a social occasion and it’s lovely. It’s very nice, yeah’. BF9 also attributed the change in OP9’s mental state to the organisation more broadly, ‘OP9 kind of turned his life around and you know, maybe ALONE played a part in that too’.

It is worth noting that some participants did not feel they had made any substantial changes to the lives of their service user by befriending. OP10 had not felt any impact of befriending on her mental health: ‘I don’t know whether she’s improved my life or not really, it’s still the same. So very hard question to answer, because I don’t think it has lifted, say my depression, in any way’. BF10 also felt that OP10 had not changed because of the service: ‘I think psychologically and physically I think that you know, the physical health issues and anxiety I think do kind of ebb and flow, so you know, I wouldn’t say she seems worse overall than when I met her so I can’t say I’d see any negative changes but I do know there are phases, if you will’. Similarly, BF3 felt that she had not caused any substantial changes in her partner: ‘I wouldn’t think she’s changed a huge amount, no. I know that she enjoys my visits and I know she looks forward to them so whether it perks her up on the day, yeah maybe, but I don’t think that’s a change’.

However, among our sample, many of whom had poor health, it is possible that a lack of decline reflects the best possible outcome, if the befriending intervention served to offset an existing decline in health.

## Discussion

We used a constructivist grounded theory approach to understanding how participants understand the ways in which befriending might impact health, and built a grounded (in the data) theoretical description of how this might occur. The current study found evidence for five potential mechanisms through which befriending may impact health, and which may contribute to our underdeveloped theoretical understanding (Lester et al., 2012). Furthermore, while prior research has focused on the impact of befriending on mental health (Fakoya et al., 2021; Mead, et al., 2010), our current findings suggest that befriending is likely to yield positive impacts on health defined more broadly. Previous research focusing on the impact of befriending on loneliness and social isolation had identified four key mechanisms: reciprocity, empathy, autonomy and privacy (Fakoya et al., 2021). The current findings overlap somewhat: provision of emotional support relies on the empathy of the befriender; but otherwise there is little overlap, suggesting that there are distinct pathways through which befriending impacts loneliness and health more broadly. Another descriptive model of befriending listed some theoretical links between befriending and health: attachment, friendship, buffering, social networks, social capital building and social inclusion (Balaam, 2015), although conceptually these overlap a little with our mechanisms. Our results also overlap somewhat with those of Gardiner on mechanisms between befriending and wellbeing; meaningful interactions and cognitive stimulation arose as potential mechanisms in their study too (Gardiner and Barnes, 2016). The mechanism we termed ‘providing emotional support’ also overlaps somewhat with ‘social support’ as a befriending mechanism impacting emotional health identified previously (Lester et al., 2012).

Results are highly compatible with Berkman’s causal cascade model of social influences on health (Berkman et al., 2000); emotional support, supporting health behaviours, cognitive stimulation, advice and opportunities for socialising all map on to the psychosocial mechanisms listed in this model. Only improvement of mood does not appear in the causal cascade model, although it could be argued that it is analogous to a sense of wellbeing (which appears in the model as a pathway, influenced by mechanisms). As such, the current findings could be taken as further validation of the causal cascade model in the context of befriending services. It would be of interest to evaluate the ‘upstream’ factors listed in the model, and how they influence befriending services in turn. Thus while the identified mechanisms have some face validity and biological plausibility, because they have to be seen as operating with complex social systems, other Bradford Hill criteria supportive of a causal warrant would be hard to demonstrate (Holt-Lunstad, 2022).

Such system factors include mezzo level factors such as the characteristics of social network ties and so it would be of interest to understand how factors such as frequency of face-to-face and organisational contact, reciprocity of ties and multiplexity all feed into the identified mechanisms in a befriending context. By the same token, we cannot claim that each mechanism would be triggered in the same way for each health outcome nor that their effects are equivalent or universal across individuals (Rieckmann et al., 2022).

It should be clarified that participants themselves did not often draw the connection between the mechanisms they described and a positive impact on health. For instance, while many reported that they provided emotional support for each other, there was no description of the impact such support could yield on health. Such impacts are evident through a synthesis of the data and prior literature on the topic identifying links between emotional support and health (Reblin and Uchino, 2008).

Findings suggest that there are multiple mechanisms through which befriending might impact health. We used a dyadic approach which extends beyond existing findings in relation to the mechanisms through which befriending impacts the older person (Krohne et al., 2023). More research is still warranted, however, to confirm the direct or overall impact of befriending on health. Currently, mixed findings exist on this impact (Siette et al., 2017), and results have been stymied by methodological limitations (Schwei et al., 2021). To attempt to contribute to this situation, we conducted a single-case experimental design evaluation of the ALONE befriending service, and demonstrated a therapeutic effect of the service on health-related quality of life of older people, alongside evidence that befriending services may act by suppressing the negative impact of loneliness on health over time. Approaches such as this may present interesting avenues for future research to tackle.

Study conclusions must be tempered with reflection on the methodological limitations of the research. We set out to conduct face-to-face dyadic interviews with the dyads, but were prevented by the COVID-19 pandemic. While we nonetheless applied a dyadic analysis to separate interview data, such dyadic data would have potentially enriched the current findings. We used constructivist grounded theory to shape the design and analysis of the study, and interpreted results using a realist evaluative definition of mechanism. It is potentially controversial to merge two distinct approaches in this manner, since we did not use realist interviews, but it is argued that the realist approach should be flexible enough to apply to other methodologies including data collected using a grounded theory approach (Kazi and Spurling, 2000). Using the dyadic approach enabled us to explore disagreement between dyad members on their reports. For instance, we found in one theme, supporting health behaviours, that while befrienders commonly described their efforts to support their partner engaging in health behaviours, only some of the older persons verified these efforts in their descriptions. It is possible that the befrienders were overestimating their role in maintaining the health of their partners, or alternatively, that the older participants were unwilling to disclose what may be perceived as dependence on their befriending partners (although some did so). Further empirical confirmatory research of the potential mechanisms identified in this study would be necessary to understand whether befrienders truly play a role in supporting health behaviours of their partners.

Notwithstanding the debate about ex ante standards for mechanistic explanation, which we believe our findings meet (Aviles and Reed, 2017), further methodological work is required to elucidate how a variety of preceding factors might combine to trigger these putative befriending mechanisms’ actions on distal health outcomes. However, we cannot claim to have given a full account of the possible mechanisms at play. For example we already observed that participants seldom drew a connection between mechanisms and health outcomes and by the same token we are mindful of ongoing work that has advanced a role for unconscious psychological processes affecting health behaviours and outcomes (Hollands et al., 2016). The extent to which these are important places limits on mechanistic reasoning in realist qualitative inquiry, limits which nevertheless may be loosened and benefit from emerging work to enhance the synergies between quantitative and qualitative methods that pursue causal inference (Drury et al., 2022; Johnson et al., 2019; Proudfoot, 2022).

A corollary of such work is that it will facilitate better theorisation and evaluation of befriending services (Bonell et al., 2023). Eventually, when the mediating or moderating roles of factors contributing to these mechanistic pathways are distinguished, we will be in a better position to tailor the interventions to those most likely to benefit (Gardner, 2023) or indeed to suggest wider societal action to enhance community social capital and social engagement (Gregorio, 2022).

In conclusion we report five potential mechanisms through which befriending services might impact on the health of its users. We suggest further quantitative research which measures the activity of such mechanisms among befriending services users to further corroborate whether they demonstrate a mechanistic action in the link between befriending and health.

## References

[bibr1-13591053241235846] Aviles NB Reed IA (2017) Ratio via machina: Three standards of mechanistic explanation in sociology. Sociological Methods & Research 46(4): 715–738.

[bibr2-13591053241235846] Balaam MC (2015) A concept analysis of befriending. Journal of Advanced Nursing 71(1): 24–34. 25318903 10.1111/jan.12553

[bibr3-13591053241235846] Bantry-White E O’Sullivan S Kenny L , et al. (2018) The symbolic representation of community in social isolation and loneliness among older people: Insights for intervention from a rural Irish case study. Health & Social Care in the Community 26(4): e552–e559. 10.1111/hsc.12569 29582501

[bibr4-13591053241235846] Beckers A Bücker S Casabianca E , et al. (2022) Effectiveness of Interventions Tackling Loneliness. EUR 31313 EN, Publications Office of the European Union, Luxembourg, 2022, ISBN 978-92-76-59108-5, doi:10.2760/277109, JRC130944.

[bibr5-13591053241235846] Berkman LF Glass T Brissette I , et al. (2000) From social integration to health: Durkheim in the new millennium. Social Science & Medicine 51: 843–857. 10972429 10.1016/s0277-9536(00)00065-4

[bibr6-13591053241235846] Bonell C Ponsford R Meiksin R , et al. (2023). Testing and refining middle-range theory in evaluations of public-health interventions: Evidence from recent systematic reviews and trials. Journal of Epidemiology and Community Health 77(3): 147–151. 36599654 10.1136/jech-2022-219776

[bibr7-13591053241235846] Bonell C Warren E Melendez-Torres G (2022) Methodological reflections on using qualitative research to explore the causal mechanisms of complex health interventions. Evaluation 28(2): 166–181.

[bibr8-13591053241235846] Breheny M Pond R Lilburn LE (2020) “What am I going to be like when I’m that age?”: How older volunteers anticipate ageing through home visiting. Journal of Aging Studies 53: 100848. 32487339 10.1016/j.jaging.2020.100848

[bibr9-13591053241235846] Burke L (2015). ALONE Befriending Service Evaluation Report. Dublin, Ireland: Liz Burke Communications.

[bibr10-13591053241235846] Cattan M White M Bond J , et al. (2005) Preventing social isolation and loneliness among older people: A systematic review of health promotion interventions. Ageing and Society 25(1): 41–67. 10.7748/nop.17.1.40.s11 27736564

[bibr11-13591053241235846] Charmaz K (2006) Constructing Grounded Theory: A Practical Guide Through Qualitative Research. London: Sage Publications Ltd.

[bibr12-13591053241235846] Dean J Goodlad R (1998) Supporting Community Participation: The Role and Impact of Befriending. Brighton,UK: Pavillion.

[bibr13-13591053241235846] Drury A Payne S Brady AM (2022) Adapting the pillar integration process for theory development: The theoretical model of healthcare factors influencing quality of life in cancer survivorship. Journal of Mixed Methods Research 17(3). DOI: 10.1177/15586898221134730

[bibr14-13591053241235846] Eisikovits Z Koren C (2010) Approaches to and outcomes of dyadic interview analysis. Qualitative Health Research 20(1): 1642–1655. 20663940 10.1177/1049732310376520

[bibr15-13591053241235846] El-Baalbaki G Arthurs E Levis B , et al. (2010) Effects of befriending on depressive symptoms: A precautionary note on promising findings. The British Journal of Psychiatry 197(3): 247–247. 20807974 10.1192/bjp.197.3.247

[bibr16-13591053241235846] Fakoya OA McCorry NK Donnelly M (2021) How do befriending interventions alleviate loneliness and social isolation among older people? A realist evaluation study. PloS One 16(9): e0256900. 10.1371/journal.pone.0256900 PMC8428774 34499682

[bibr17-13591053241235846] Gardiner C Barnes S (2016) The impact of volunteer befriending services for older people at the end of life: Mechanisms supporting wellbeing. Progress in Palliative Care 24(3): 159–164.

[bibr18-13591053241235846] Gardiner C Geldenhuys G Gott M (2018) Interventions to reduce social isolation and loneliness among older people: An integrative review. Health and Social Care in the Community 26(2): 147–157. 27413007 10.1111/hsc.12367

[bibr19-13591053241235846] Gardner F (2023) Commentary for special issue on using baseline target moderation to assess variation in prevention impact: When (and how) to revise our programs. Prevention Science 24(2): 299–303. 36418802 10.1007/s11121-022-01458-1 PMC9938011

[bibr20-13591053241235846] Glaser BG Strauss AL (2009) The Discovery of Grounded Theory: Strategies for Qualitative Research. Chicago, USA: Transaction publishers.

[bibr21-13591053241235846] Godfrey M (2015) Qualitative Research in Age and Ageing: Enhancing Understanding of Ageing, Health and Illness. Age and Ageing 44(5): 726–727. 26209785 10.1093/ageing/afv096

[bibr22-13591053241235846] Golden J Conroy RM Lawlor BA (2009) Social support networks in older people: Underlying dimensions and association with psychological and physical health. Psychology Health & Medicine 14(3): 280–290. 10.1080/13548500902730135 19444706

[bibr23-13591053241235846] Gregorio DI (2022) Precursory prevention: Togetherness for better health. American Journal of Preventive Medicine 63(4): 656–659. 35780005 10.1016/j.amepre.2022.04.029 PMC9242725

[bibr24-13591053241235846] Hollands GJ Marteau TM Fletcher PC (2016). Non-conscious processes in changing health-related behaviour: A conceptual analysis and framework. Health Psychology Review 10(4): 381–394. 26745243 10.1080/17437199.2015.1138093 PMC5214381

[bibr25-13591053241235846] Holt-Lunstad J (2022) Social connection as a public health issue: The evidence and a systemic framework for prioritizing the “social” in social determinants of health. Annual Review of Public Health 43: 193–213. 10.1146/annurev-publhealth-052020-110732 35021021

[bibr26-13591053241235846] Hunter A Keady J Casey D , et al. (2016) Psychosocial intervention use in long-stay dementia care: A classic grounded theory. Qualitative Health Research 26(14): 2024–2034. 26935720 10.1177/1049732316632194

[bibr27-13591053241235846] Johnson RB Russo F Schoonenboom J (2019) Causation in mixed methods research: The meeting of philosophy, science, and practice. Journal of Mixed Methods Research 13(2): 143–162.

[bibr28-13591053241235846] Jopling K (2015) Promising Approaches to Reducing Loneliness and Social Isolation in Later Life. London, UK: Age UK.

[bibr29-13591053241235846] Kazi MA Spurling LJ (2000) Realist evaluation for evidence-based practice. Paper presented at the 2000 European Evaluation Society Conference, 12–14 October, Lausanne, Switzerland.

[bibr30-13591053241235846] Koren C (2016) Men’s vulnerability – women’s resilience: From widowhood to late-life repartnering. International Psychogeriatrics 28(5): 719–731. 26691683 10.1017/S1041610215002240

[bibr31-13591053241235846] Krohne K Flor AD Nicolaisen M (2022) Friendship in befriending? Older service users’ notions of friendship in a befriending scheme. Journal of Gerontological Social Work 66(4): 459–473. 36052456 10.1080/01634372.2022.2118406

[bibr32-13591053241235846] Krohne K Flor AD Nicolaisen M (2023) Friendship in befriending? Older service users’ notions of friendship in a befriending scheme. Journal of Gerontological Social Work 66(4): 459–473. 36052456 10.1080/01634372.2022.2118406

[bibr33-13591053241235846] Lester H Mead N Graham CC , et al. (2012) An exploration of the value and mechanisms of befriending for older adults in England. Ageing & Society 32(2): 307–328.

[bibr34-13591053241235846] Levitt HM Bamberg M Creswell JW , et al. (2018) Journal article reporting standards for qualitative primary, qualitative meta-analytic, and mixed methods research in psychology: The APA publications and communications board task force report. American Psychologist 73(1): 26–46. 29345485 10.1037/amp0000151

[bibr35-13591053241235846] Lincoln YS (2005) The Sage Handbook of Qualitative Research. University of Illinois, Urbana - Champaign; Sage.

[bibr36-13591053241235846] Malterud K Siersma VD Guassora AD (2016) Sample size in qualitative interview studies: Guided by information power. Qualitative Health Research 26(13): 1753–1760. 26613970 10.1177/1049732315617444

[bibr37-13591053241235846] Mead N Lester H Chew-Graham C , et al. (2010) Effects of befriending on depressive symptoms and distress: Systematic review and meta-analysis. The British Journal of Psychiatry 196(2): 96–101. 20118451 10.1192/bjp.bp.109.064089

[bibr38-13591053241235846] Moore G Audrey S Barker M (2014) Process Evaluation of Complex Interventions: UK Medical Research Council (MRC) Guidance. London: MRC Population Health Science Research Network.

[bibr39-13591053241235846] Moss B Behn N Northcott S , et al. (2021) “Loneliness can also kill:” A qualitative exploration of outcomes and experiences of the SUPERB peer-befriending scheme for people with aphasia and their significant others. Disability and Rehabilitation 44(18): 5015–5024. 34086521 10.1080/09638288.2021.1922519

[bibr40-13591053241235846] Pitkala KH Routasalo P Kautiainen H , et al. (2011) Effects of socially stimulating group intervention on lonely, older people’s cognition: A randomized, controlled trial. The American Journal of Geriatric Psychiatry 19(7): 654–663. 21709611 10.1097/JGP.0b013e3181f7d8b0

[bibr41-13591053241235846] Pitkala KH Routasalo P Kautiainen H , et al. (2009) Effects of psychosocial group rehabilitation on health, use of health care services, and mortality of older persons suffering from loneliness: A randomized, controlled trial. Journals of Gerontology Series A: Biomedical Sciences and Medical Sciences 64(7): 792–800. 10.1093/gerona/glp011 19223606

[bibr42-13591053241235846] Proudfoot K (2022) Inductive/Deductive hybrid thematic analysis in mixed methods research. Journal of Mixed Methods Research 2003: 308–326.

[bibr43-13591053241235846] Reblin M Uchino BN (2008) Social and emotional support and its implication for health. Current Opinion in Psychiatry 21(2): 201. 18332671 10.1097/YCO.0b013e3282f3ad89 PMC2729718

[bibr44-13591053241235846] Rieckmann A Dworzynski P Arras L , et al. (2022) Causes of Outcome Learning: A causal inference-inspired machine learning approach to disentangling common combinations of potential causes of a health outcome. International Journal of Epidemiology 51(5): 1622–1636. 35526156 10.1093/ije/dyac078 PMC9799206

[bibr45-13591053241235846] Routasalo PE. Tilvis RS. Kautiainen H , et al. (2009) Effects of psychosocial group rehabilitation on social functioning, loneliness and well-being of lonely, older people: Randomized controlled trial. Journal of Advanced Nursing 65(2): 297–305. 19054177 10.1111/j.1365-2648.2008.04837.x

[bibr46-13591053241235846] Schwei RJ Hetzel S Kim K , et al. (2021) Peer-to-peer support and changes in health and well-being in older adults over time. JAMA Network Open 4(6): e2112441–e2112441. 10.1001/jamanetworkopen.2021.12441 PMC8207241 34129024

[bibr47-13591053241235846] Siette J Cassidy M Priebe S (2017) Effectiveness of befriending interventions: A systematic review and meta-analysis. BMJ Open 7: e014304. 10.1136/bmjopen-2016-014304 PMC5594212 28446525

[bibr48-13591053241235846] Sikira H Janković S Slatina MS , et al. (2021) The effectiveness of volunteer befriending for improving the quality of life of patients with schizophrenia in Bosnia and Herzegovina–An exploratory randomised controlled trial. Epidemiology and Psychiatric Sciences 30: e48. 10.1017/S2045796021000330 PMC8220484 34112279

[bibr49-13591053241235846] Tong A Sainsbury P Craig J (2007) Consolidated criteria for reporting qualitative research (COREQ): A 32-item checklist for interviews and focus groups. International Journal for Quality in Healthcare 19(6): 349–357. 10.1093/intqhc/mzm042 17872937

[bibr50-13591053241235846] World Health Organization (1995) Constitution of the World Health Organization. Basic Documents. Geneva: World Health Organization.

[bibr51-13591053241235846] Yu H Liu J (2010) A qualitative study of exploring the therapeutic components of traditional Chinese medicine as a complex intervention through grounded theory. Journal of Chinese Integrative Medicine 8(10): 928–943. 20939983 10.3736/jcim20101004

